# A call for principle-based acceptance of synthetic patients: establishing the AI-driven foundation for regulatory submissions

**DOI:** 10.3389/fdgth.2026.1833779

**Published:** 2026-07-24

**Authors:** Jordi Guitart

**Affiliations:** M47 Labs, Barcelona, Spain

**Keywords:** clinical trials, drug development, GANs, generative AI, rare diseases, regulatory framework, synthetic patient data, VAEs

## Abstract

The pharmaceutical industry stands at the precipice of an AI-driven data revolution, with synthetic patients emerging as a transformative tool to accelerate drug discovery and development while enhancing patient privacy. However, a critical regulatory gap persists: the absence of a standardized basis from leading regulatory bodies for accepting AI-generated patient populations as evidence in regulatory submissions. This manuscript addresses this void by proposing five foundational principles—Representativeness, Utility, Robustness, Privacy Preservation, and Transparency—anchored by the “Fit for Purpose” philosophy. We introduce the operational concept of a “Technical Validation Playbook” to facilitate the first wave of regulatory acceptances for synthetic patient data. We further outline actionable recommendations for regulatory agencies and pharmaceutical sponsors to advance the acceptance of synthetic patient populations through existing qualification and scientific advice mechanisms. By establishing a proactive, principle-based approach, this framework aims to catalyze regulatory-industry alignment and unlock the transformative potential of synthetic patients, particularly for populations with unmet medical needs such as rare diseases where traditional placebo-controlled trials face insurmountable ethical and recruitment challenges.

## 1 Introduction

The established regulatory landscape for pharmaceutical development, governed by International Council for Harmonisation (ICH) guidelines and Good Clinical Practice (GCP), does not explicitly address the generation, validation, and regulatory acceptance of synthetic patients derived from generative artificial intelligence models. While progressive updates such as ICH E6(R3) embrace contemporary data management approaches, they primarily focus on clinical trials involving real human participants (1). Similarly, ISO/IEC 42001:2023 addresses AI management systems broadly but lacks specific parameters for synthetic clinical data construction and validation (2).

Recent regulatory initiatives signal growing recognition of AI's potential in pharmaceutical development. The U.S. Food and Drug Administration's (FDA) 2025 draft guidance on artificial intelligence for regulatory decision-making represents a significant milestone toward structured oversight (3). However, this guidance does not provide explicit acceptance criteria for synthetic patient populations generated through data-driven generative models such as Generative Adversarial Networks (GANs) or Variational Autoencoders (VAEs). As confirmed by recent comprehensive reviews, no specific recommendations have been outlined for synthetic data or generative AI models in pharmaceutical applications (4, 5). This stands in contrast to the maturing regulatory engagement with AI/ML-enabled medical devices, of which the FDA had authorized over 1,000 as of December 2024—illustrating that regulatory mechanisms can evolve to accommodate AI once fit-for-purpose evaluation criteria exist.

Throughout this manuscript, we use the term synthetic patient generation to denote the creation of complete, non-real patient records—structured tabular data of the kind captured in the electronic case report form—intended to serve as patient cohorts within clinical-trial evidence. We distinguish this from the broader category of synthetic data generation, which also encompasses medical imaging, free-text clinical notes, and the augmentation of real-world data. The framework presented here addresses synthetic patient generation on structured tabular data, the modality on which current regulatory decision-making depends (Section 3.3).

While computational modeling and simulation (such as physiologically based pharmacokinetic models) have established regulatory precedent, no standardized basis yet exists for accepting AI-generated synthetic patient cohorts as primary or supportive evidence in drug development (6, 7). This is not for want of regulatory engagement with synthetic-data quality: the FDA's Center for Devices and Radiological Health has proposed a multi-criteria evaluation framework and reporting scorecard for synthetic medical data (8), and the UK MHRA has published principle-based considerations for synthetic data in the development of AI as a medical device (9, 10). These instruments evaluate and report data quality and extend principle-based expectations to synthetic data, including its use in clinical trials for medicines; they do not, however, establish how generated synthetic patient cohorts should be accepted as substantive clinical evidence. The present framework addresses that distinct question. This gap remains consequential given that no drug or medical device has been registered using predominantly synthetic data as primary evidence (11).

The urgency for regulatory clarity intensifies as pharmaceutical companies increasingly partner with AI-driven biotech firms capable of generating high-fidelity synthetic patient cohorts. Without standardized validation principles, sponsors face uncertainty regarding evidentiary sufficiency, potentially delaying innovations that could benefit patients with limited treatment options.

## 2 Principle-based framework for regulatory acceptance

In the absence of prescriptive regulations, we propose that the “Fit for Purpose” principle must serve as the guiding philosophy: synthetic patient data must be demonstrably adequate, appropriate, and reliable for its intended regulatory use. Building upon this foundation, we present five core principles designed to establish regulatory confidence and facilitate evidence-based evaluation:

### 2.1 Representativeness (fidelity)

The synthetic population must capture the essential statistical characteristics, clinical heterogeneity, and biological plausibility of the real-world target population. This extends beyond univariate distributional similarity to encompass multivariate dependencies that reflect authentic disease biology and treatment responses.

### 2.2 Utility (value proposition)

Synthetic patients must serve demonstrable regulatory and clinical purposes that cannot be adequately addressed through conventional approaches. Valid use cases include: (i) augmenting limited patient populations in rare diseases where recruiting large numbers of participants is infeasible; (ii) generating diverse demographic representations to address health disparities; (iii) privacy-preserving data sharing for collaborative research; and (iv) training and validating AI/ML diagnostic or prognostic models (12, 13).

### 2.3 Robustness (reliability and consistency)

The generative process must be reproducible, algorithmically stable, and methodologically transparent. Validation results should demonstrate consistency across multiple independent synthetic cohorts generated from the same source data, ensuring that observed fidelity is not artifactual.

### 2.4 Privacy preservation (data protection)

From a medical-products regulatory perspective, privacy preservation is evaluated chiefly in relation to its effect on utility: privacy-protective measures must not degrade the fidelity required for the intended regulatory use. Regulatory positions on privacy during generation differ—an emphasis on fidelity with disclosure risk managed through secure access, vs. the inclusion of formal privacy metrics with region-adjustable thresholds—so the appropriate treatment is context-dependent. We therefore frame privacy preservation as disclosure-risk quantification and control coupled to utility, and recommend that sponsors report the privacy–utility trade-off explicitly rather than optimizing either objective in isolation.

Synthetic data must be demonstrably free from re-identification risks. This requires rigorous privacy stress-testing using established attack models and, where applicable, formal privacy guarantees such as differential privacy (14–16). Sponsors must provide quantitative evidence that synthetic patients cannot be reverse-engineered to reveal identifiable information about real individuals.

### 2.5 Transparency (documentation)

The complete lifecycle—from source data provenance to model architecture, training procedures, hyperparameters, and validation methodologies—must be comprehensively documented. This transparency enables independent regulatory review and verification of claims regarding fidelity, utility, and privacy.

### 2.6 Epistemological justification for principle-based validation

The necessity for this principle-based framework arises from the inherent complexity of modern generative AI architectures. Advanced models such as GANs and VAEs operate as high-dimensional transformations where the mapping from latent space to synthetic output lacks straightforward interpretability (17–20). Unlike mechanistic simulation models where assumptions can be verified equation-by-equation, these data-driven models function as learned approximations of complex probability distributions.

Consequently, when pharmaceutical sponsors present generative models to regulators, certainty cannot be established through traditional code audits or algorithmic transparency alone. The “black box” nature of deep neural networks necessitates an empirical approach: regulatory confidence must be derived from rigorous demonstration of equivalence between real and synthetic populations. By proving absolute Representativeness through systematic validation, sponsors provide empirical evidence that—despite the model's opacity—the generated data constitutes a faithful and reliable proxy for biological reality.

This epistemological shift from mechanistic verification to empirical validation represents a fundamental adaptation in regulatory science for the AI era. These five principles can be situated against the evaluation criteria that regulators have begun to articulate for synthetic data; Table 1 provides a crosswalk between the framework proposed here and the FDA and MHRA criteria.

**Table 1:** Crosswalk between the five foundational principles and existing regulatory evaluation criteria for synthetic medical data.

Five foundational principles	FDA scorecard criteria (8)	MHRA-associated criteria (9, 10)
Representativeness	Coverage (variability, range and novelty against true distributions); Congruence (distributional alignment with real data); Consistency (across demographic and disease subgroups)	Representativeness; Fidelity
Utility	Addressed through task-based evaluation rather than a single named criterion; Completeness (information needed for the intended use) and Constraint (clinically valid data points) act as contributing attributes of fitness for the intended analytic purpose	Utility (fitness for the intended analytic purpose)
Robustness	Consistency; Constraint (stability and clinical validity across cases)	No dedicated criterion; addressed within fidelity assessment and the report's reflective questions
Privacy Preservation	Compliance (de-identification, privacy preservation and adherence to standards)	Addressed primarily through secure access rather than generation-time privacy measures
Transparency	Comprehension (accessibility and clarity of generation processes and documentation)	Transparency

1. The mapping shows close alignment on representativeness and transparency, differing approaches to privacy (see Note 3), and identifies criteria and a principle that lack direct cross-framework analogues.

2. The FDA criteria Constraint and Completeness map only partially onto the five principles (chiefly Utility and Robustness) and have no dedicated counterpart, reflecting the scorecard's broader, modality-spanning scope. Robustness, conversely, has no direct analogue among the MHRA-associated criteria.

3. Agencies diverge on privacy: the FDA scorecard incorporates formal privacy metrics under Compliance, whereas the MHRA-associated sources prioritize fidelity and manage disclosure risk through secure access. The present framework treats privacy as context- and region-dependent (Section 2.4).

4. FDA criteria are drawn from the synthetic medical data scorecard (8); MHRA-associated criteria from the AIaMD regulatory considerations (9), and the data-quality perspective chapter (10).

## 3 Operationalizing the framework: the technical validation playbook

The transition from theoretical AI capabilities to admissible regulatory evidence requires a standardized pathway for submission and evaluation. Given that no clinical trials have yet been validated using AI-generated synthetic data as primary or supportive evidence, we propose an operational construct to bridge this evidentiary gap.

### 3.1 The technical validation playbook

To satisfy the “Fit for Purpose” requirement and operationalize the five principles, sponsors should prepare a structured, three-tiered Technical Validation Playbook for regulatory submission. This playbook serves as the evidentiary foundation for demonstrating that synthetic patient populations meet regulatory standards for their intended use. The Technical Validation Playbook comprises:

#### 3.1.1 Tier 1: seed data baseline

Comprehensive documentation of the real-world source data used to train the generative AI models. This section must establish that the foundation of the synthetic population is grounded in verified clinical reality. Required elements include documentation of data provenance and collection methodology, the patient selection criteria and inclusion–exclusion logic applied, the demographic and clinical characteristics of the source cohort, data quality assessments and preprocessing procedures, and regulatory compliance documentation such as ethics approvals and consent provisions for secondary data use.

#### 3.1.2 Tier 2: synthetic dataset

The generated population demonstrating high multivariate fidelity to the source data. This tier provides the synthetic cohort proposed for the regulatory use case (e.g., an external control arm or a supplementary population for subgroup analysis). Sponsors should provide complete synthetic patient records in structured format, descriptive statistics demonstrating distributional similarity, clinical plausibility assessments conducted by domain experts, and a justification of the synthetic cohort sample size.

#### 3.1.3 Tier 3: validation evidence Set

A rigorous technical report detailing validation methodologies, performance benchmarks, and privacy assessments. This tier operationalizes the five principles through quantitative evidence.

##### 3.1.3.1 Representativeness

Validation should include univariate distributional comparisons using appropriate statistical tests, multivariate fidelity assessments based on global distance metrics, and clinical coherence evaluations conducted by medical experts.

##### 3.1.3.2 Utility

Validation should demonstrate performance under the train-on-synthetic-test-on-real (TSTR) paradigm (21, 22), provide use-case–specific evidence that the synthetic data achieve their intended analytical purpose and include comparative analyses showing non-inferiority to real data for the target application.

##### 3.1.3.3 Robustness

Validation should include reproducibility evidence across multiple model training runs, sensitivity analyses assessing stability under hyperparameter variations, cross-validation results demonstrating consistent performance, and triangulation-based validation comparing synthetic cohorts generated by different model families to mitigate architecture-specific biases.

##### 3.1.3.4 Privacy preservation

Validation should include quantitative privacy risk assessments using disclosure-risk metrics, results from privacy attack simulations such as membership inference or attribute disclosure, and documentation of the differential privacy budget when formal privacy guarantees are employed (14–16).

##### 3.1.3.5 Transparency

Validation should include complete model specification covering architecture, loss functions, and training procedures, together with documentation of hyperparameter settings and optimization strategies, availability of source code or sufficiently detailed algorithmic descriptions, and an explicit statement of limitations and known biases.

**Figure 1:**
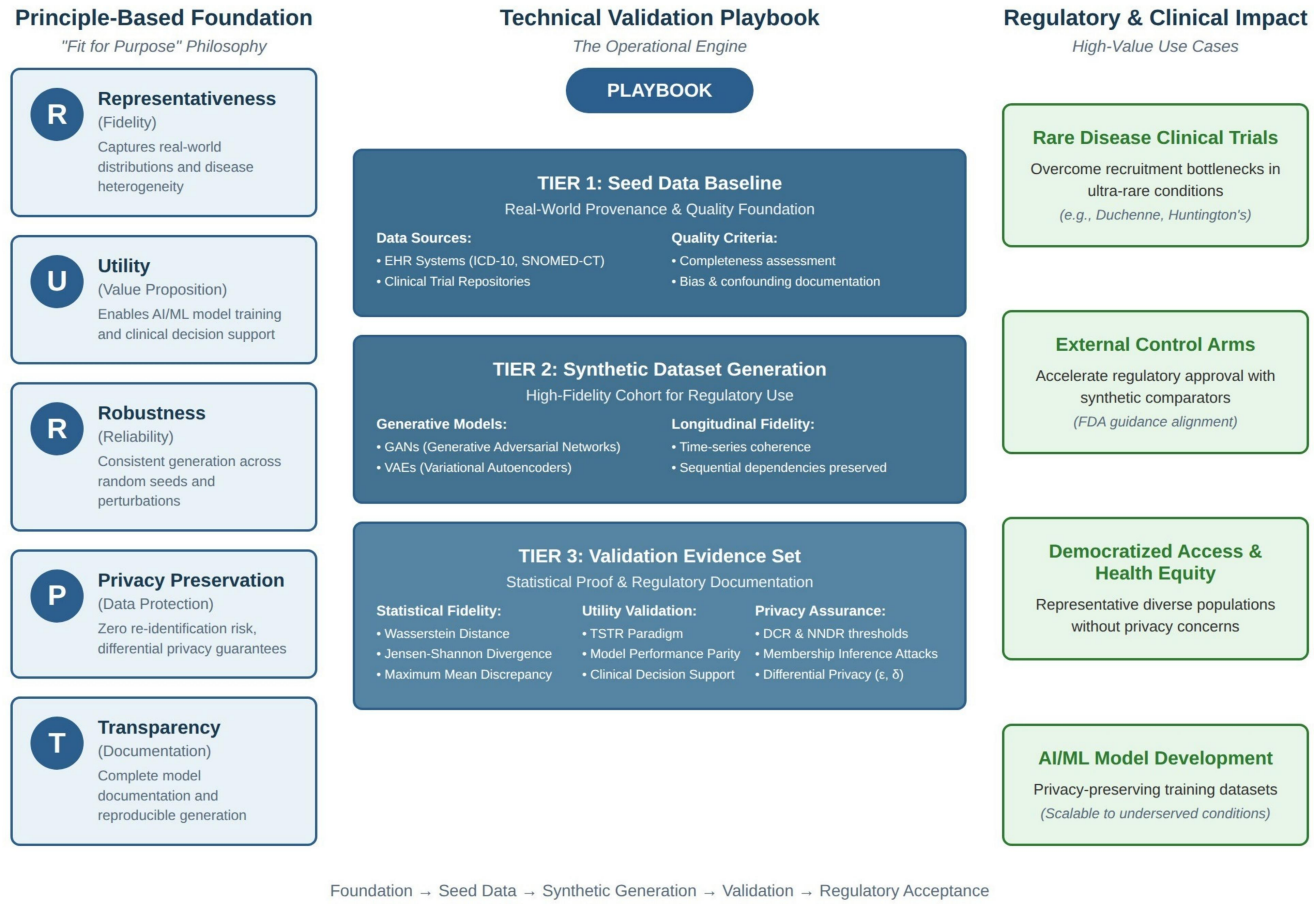
Technical validation playbook architecture. This schematic illustrates the proposed Technical Validation Playbook, an operational framework designed to address the evidentiary gap in accepting synthetic clinical data as regulatory evidence. **Principle-Based Foundation:** The framework is anchored by the “Fit for Purpose” philosophy and five core principles: Representativeness, Utility, Robustness, Privacy Preservation, and Transparency. **Technical Validation Playbook:** Tier 1, Seed Data Baseline: Establishes the clinical reality of the source cohort, including data provenance, quality assessments, and ethical compliance. Tier 2, Synthetic Dataset Generation: Details the generation of high-fidelity cohorts specifically intended for regulatory use cases, such as external control arms or subgroup analyses. Tier 3, Validation Evidence Set: Operationalizes the five principles through quantitative benchmarks, including global distance metrics (e.g., Wasserstein Distance) for fidelity, the Train-on-Synthetic-Test-on-Real (TSTR) paradigm for utility, and empirical privacy stress-testing. **Regulatory Outcomes:** The Playbook facilitates structured evidence-based evaluation, particularly for rare disease trials and populations where traditional placebo-controlled methodologies are ethically or practically constrained. DCR, Distance to Closest Record; NNDR, Nearest Neighbor Distance Ratio; GANs, Generative Adversarial Networks; TSTR, Train-on-Synthetic-Test-on-Real; VAEs, Variational Autoencoders; EHR, Electronic Health Record; ICD-10, International Classification of Diseases 10th Revision; SNOMED-CT, Systematized Nomenclature of Medicine—Clinical Terms.

This structured approach ensures that regulatory evaluators have access to comprehensive evidence supporting each principle, facilitating systematic and objective assessment (see Figure 1).

### 3.2 Quantitative validation methodologies

The validation of synthetic patient data requires both statistical rigor and clinical meaningfulness. We recommend the following methodological standards:

#### 3.2.1 Statistical validation for representativeness

##### 3.2.1.1 Univariate assessments

For continuous variables, the two-sample Kolmogorov–Smirnov (K-S) test provides non-parametric comparison of cumulative distribution functions between real and synthetic data (23). For categorical variables, Chi-square tests or Fisher's exact tests assess frequency distribution concordance.

##### 3.2.1.2 Multivariate assessments

Global distributional distance metrics are required to capture joint dependencies that cannot be detected through univariate tests alone. Recommended approaches include the Wasserstein distance (Earth Mover's Distance), which quantifies the minimum “cost” required to transform one distribution into another (12, 24); maximum mean discrepancy (MMD), a kernel-based metric that compares distributions in high-dimensional feature spaces and is sensitive to differences in correlation structures (25); and Jensen–Shannon divergence, a bounded and symmetric measure of similarity between probability distributions (26). Together, these multivariate metrics help ensure that synthetic datasets preserve clinically meaningful interdependencies—such as the relationship between age, comorbidities, and disease severity—that are critical for valid external control applications.

#### 3.2.2 Utility validation: TSTR paradigm

The “Train-on-Synthetic-Test-on-Real” (TSTR) framework provides direct evidence of utility (21, 22). In this approach, a predictive model is first trained exclusively on synthetic data, after which its performance is evaluated on held-out real data. The resulting performance is then compared with that of a benchmark model trained directly on real data.

If the TSTR model achieves comparable performance (within pre-specified margins), this demonstrates that synthetic data capture the predictive structure necessary for the intended application. This paradigm is particularly relevant for validating synthetic data intended to train diagnostic or prognostic AI/ML models (27).

An important scope condition applies to TSTR validation. The paradigm presupposes that the synthetic data are intended to reproduce the informational content of an existing source population—the reproductive, privacy-preserving regime. Where synthetic patients are instead generated to address a gap in the source data, such as representing demographic subgroups or rare phenotypes not present in the seed cohort, TSTR cannot establish non-inferiority, because no corresponding real-world observations exist for comparison. In such extrapolative scenarios, validity must instead be argued through mechanistic or clinical plausibility assessment, external benchmarking against independent real datasets where available, and explicit, auditable documentation of the extrapolation assumptions and their clinical justification. The evidentiary burden is correspondingly higher for extrapolative than for reproductive synthetic data, and regulatory expectations should be calibrated accordingly.

#### 3.2.3 Privacy risk quantification

Privacy assessment must go beyond theoretical claims to empirical testing. Recommended approaches include the Distance to Closest Record (DCR) and the Nearest Neighbor Distance Ratio (NNDR), which measure the minimum distance and relative proximity between each synthetic record and all real records to detect potential memorization or insufficient diversity in the generative output (22, 28). Additional evaluation should include membership inference attacks, which attempt to determine whether specific real patients were included in the training dataset and therefore quantify potential information leakage (29), as well as attribute disclosure risk, which assesses whether adversaries can infer sensitive attributes of real patients from the synthetic data (30). Acceptance thresholds should reference established regulatory guidance; for example, disclosure risk below 9% has been suggested by Health Canada and the European Medicines Agency for certain applications (31, 32).

### 3.3 Multimodal validation: structured vs. unstructured data

A critical distinction must be drawn between the data modalities that currently govern regulatory decision-making and those that remain under active research development. In contemporary clinical trials, the electronic case report form (eCRF) constitutes the definitive evidentiary instrument upon which regulators base progression decisions across all trial phases, including marketing authorization. The eCRF is a structured, auditable tabular dataset—comprising demographic, clinical, laboratory, and outcome variables—that serves as the regulatory ground truth irrespective of the richer data sources available upstream in the clinical workflow. The transcription of clinical observations from electronic health records into the eCRF, typically performed by clinical research organizations, represents the established interface between clinical practice and regulatory evidence. This operational reality has a direct implication for the scope and prioritization of synthetic patient validation: structured tabular data are not merely one modality among several but the modality upon which regulatory acceptance fundamentally depends. The validation methodologies proposed in this framework are accordingly designed to address this primary modality with full rigor. The discussion of unstructured data validation that follows is included to provide methodological guidance for future applications as the clinical trial ecosystem evolves but should be understood as forward-looking guidance for an evolving regulatory landscape.

#### 3.3.1 Structured tabular data

For clinical databases containing demographic, laboratory, vital signs, and outcome data, mathematical validation is appropriate and sufficient. The quantitative metrics described in Section 3.2 enable objective assessment of distributional similarity, predictive utility, and privacy preservation. Validation should proceed through a structured protocol that includes applying univariate statistical tests to each variable, calculating global distance metrics to evaluate the joint distribution, executing Train-on-Synthetic-Test-on-Real (TSTR) benchmarking for the intended use case, performing quantitative privacy risk assessments, and documenting all results within Tier 3 of the Technical Validation Playbook.

The framework is method-agnostic. While GANs and VAEs are widely used, Bayesian-network and diffusion-based approaches may be preferable for the small source cohorts typical of rare-disease settings, where adversarial models are more prone to bias amplification and to omission of distribution tails. The choice of generator is itself a fit-for-purpose decision to be justified within Tier 1.

#### 3.3.2 Unstructured data (medical imaging and clinical text)

For modalities such as medical imaging (MRI, CT, x-ray) or free-text clinical notes, mathematical distance metrics are necessary but insufficient to guarantee clinical safety and diagnostic validity. AI-generated images may exhibit statistical similarity to real images while containing subtle artifacts capable of misleading clinical interpretation or introducing spurious diagnostic patterns (33, 34).

Human-in-the-Loop (HITL) validation protocol. Sponsors should therefore provide evidence of expert review demonstrating that synthetic data are clinically indistinguishable from authentic data and free from AI-generated artifacts. For medical images, this validation should include blinded evaluation by board-certified radiologists or relevant specialists, Turing-test–style assessments in which experts attempt to distinguish real from synthetic images, systematic review for common generative artifacts such as anatomical inconsistencies or unrealistic textures, and confirmation that synthetic images contain diagnostically meaningful features without introducing misleading patterns.

For clinical text, validation should involve review by experienced clinicians to assess medical plausibility, linguistic evaluation to ensure natural language coherence, verification that synthetic notes capture appropriate clinical reasoning patterns, and assessment for potentially harmful medical inaccuracies or internal contradictions.

This multimodal validation approach recognizes that high-stakes clinical applications require safeguards beyond computational metrics when human interpretation and decision-making are involved.

### 3.4 Longitudinal vs. cross-sectional synthetic data

The choice between longitudinal and cross-sectional synthetic data generation represents a strategic decision with implications for utility, complexity, and privacy risk (15, 35).

#### 3.4.1 Utility-representativeness trade-offs

##### 3.4.1.1 Longitudinal synthetic data

These datasets are essential for applications that require temporal dynamics and simulation of patient trajectories, including disease progression modeling (36, 37), prediction of treatment response over time, digital twin applications for personalized medicine (38), and comparative effectiveness research that relies on follow-up data. Longitudinal generative models (e.g., time-aware GANs or recurrent VAEs) must therefore preserve sequential dependencies in which past clinical events influence future states. This capability enables realistic simulation of evolving patient characteristics and captures clinically meaningful phenomena such as disease trajectories, treatment switching, and cumulative adverse events (35, 39).

##### 3.4.1.2 Cross-sectional synthetic data

In contrast, cross-sectional synthetic datasets are appropriate for applications requiring snapshot representations without temporal dependencies. Typical use cases include augmenting sample size for subgroup analyses, generating diverse populations for algorithmic fairness testing, enabling privacy-preserving data sharing in collaborative model development, and constructing external control arms when only baseline and endpoint data are required.

#### 3.4.2 Privacy preservation considerations

The temporal dimension of synthetic datasets introduces distinct privacy challenges (15, 40, 41). In longitudinal data, sequential clinical patterns can function as quasi-identifiers—for example, unique treatment sequences or rare event trajectories that may allow individual identification. Longitudinal linkage attacks may exploit temporal correlations across records, and preserving realistic temporal dependencies can therefore conflict with strong privacy guarantees.

In contrast, cross-sectional synthetic datasets offer several privacy advantages. By eliminating temporal connectivity, they inherently reduce the risk of re-identification because each synthetic observation represents an independent snapshot rather than part of a patient trajectory. This independence also simplifies privacy analysis by removing sequential dependencies that could otherwise enable trajectory-based inference attacks.

These considerations carry important regulatory implications. Sponsors proposing longitudinal synthetic datasets should provide enhanced privacy evidence, potentially including formal differential privacy guarantees with documented privacy budgets, longitudinal-specific privacy attack assessments, sensitivity analyses demonstrating that rare temporal patterns cannot uniquely identify individuals, and justification that preservation of longitudinal structure is essential for the intended use case. When cross-sectional data are sufficient for the research objective, this simpler design is generally preferable from both privacy and validation-complexity perspectives.

## 4 Actionable recommendations

### 4.1 Existing regulatory mechanisms that enable adoption

Although AI-generated synthetic patient data currently lack explicit regulatory precedent, established principles from adjacent methodological domains provide useful guidance. Process-driven simulation approaches—such as physiologically based pharmacokinetic (PBPK) modeling and in silico clinical trials using mechanistic models—have progressively gained regulatory acceptance through demonstration of biological validity and predictive accuracy (42, 43). These precedents illustrate that computationally generated patient data can inform regulatory decision-making when supported by robust validation evidence.

A terminological distinction is warranted here. Mechanistic in silico methods—such as PBPK models and mechanistic in silico trials—generate data from explicit physiological equations, whereas AI-generated synthetic patient data are produced by data-driven generative models that learn distributions from real source data. These are methodologically distinct, and we invoke the former not as an equivalent technology but as an instructive regulatory precedent for how computationally generated evidence can gain acceptance through rigorous, fit-for-purpose validation.

Additional conceptual grounding can be found in recent regulatory guidance on external control arms, which allow historical or real-world data to serve as comparators in clinical studies (44, 45). Synthetic patient populations may be understood as a natural extension of this concept, whereby external data are systematically generated rather than retrospectively assembled. The core validation principles applied to external control arms—including comparability, contemporary relevance, and bias mitigation—translate directly to the evaluation of synthetic cohorts.

Regulatory agencies have also demonstrated a willingness to accommodate methodological innovation through qualification procedures, scientific advice mechanisms, and adaptive regulatory pathways (46). The Technical Validation Playbook proposed in this work aligns with these mechanisms by providing a structured evidentiary framework for regulatory evaluation of synthetic data methodologies.

### 4.2 Early regulatory dialogue as a prerequisite

As immediate first steps, sponsors should seek early regulatory dialogue through existing mechanisms. Early scientific advice consultations—such as interactions with the Innovation Task Force of the European Medicines Agency or pre-submission meetings with the U.S. Food and Drug Administration—can help establish case-specific acceptance criteria prior to formal regulatory submission (47). Through iterative dialogue, these interactions can refine the principles and validation standards proposed here and contribute to the gradual development of formal regulatory guidance for synthetic patient methodologies.

### 4.3 A roadmap toward regulatory integration

Translating this framework into regulatory practice will require coordinated progress across multiple stakeholder groups. To realize these principles in practice, the following milestones should be pursued: (i) regulatory agencies issue qualification opinions for specific synthetic data generators within a clearly defined context of use—not as blanket endorsements, but in the same context-bound manner in which existing qualification mechanisms already apply to other novel methodologies, with fitness for purpose assessed case by case; (ii) pharmaceutical sponsors submit synthetic control arms as part of hybrid clinical trial designs, establishing regulatory precedent through the first successful approvals; (iii) international initiatives—such as those coordinated through the International Council for Harmonisation (ICH)—develop consistent standards for the acceptance of synthetic patient data; and (iv) patient advocacy organizations recognize synthetic data as a pathway to accelerating access to treatments for neglected and rare conditions.

### 4.4 Operational mechanisms for multi-stakeholder implementation

Structured dialogue between industry and regulatory authorities will be essential. Progress will depend on the establishment of multi-stakeholder working groups that bring together regulatory scientists, pharmaceutical statisticians, AI researchers, and patient representatives to refine acceptance criteria through real-world case studies. Regulatory agencies could also initiate pilot programs that provide expedited review pathways for sponsors proposing synthetic data applications in rare disease trials, using these initiatives as learning opportunities to build an empirical evidence base. This direction is consistent with current regulatory momentum: in April 2026 the FDA issued a request for information on a proposed pilot program for AI-enabled optimization of early-phase clinical trials, explicitly prioritizing rare-disease trials and limited patient populations (48). While that initiative concerns AI-enabled trial optimization rather than synthetic patient cohorts specifically, it signals institutional readiness for the structured, pilot-based engagement this framework advocates. At the same time, pre-competitive collaboration among industry consortia could facilitate the sharing of validation methodologies and benchmarking results, helping to establish best practices and elevate quality standards across the field. Finally, coordinated international efforts involving agencies such as the U.S. Food and Drug Administration, the European Medicines Agency, the Pharmaceuticals and Medical Devices Agency, and other regulators will be necessary to prevent fragmented requirements that could impede global drug development.

The principles and operational framework proposed herein provide a structured foundation upon which regulatory agencies and sponsors can begin building case-specific acceptance pathways. Realizing this vision, however, will require coordinated action. Achieving these outcomes will require sustained investment in validation by sponsors, scientifically grounded flexibility from regulators, and a shared commitment across stakeholders to prioritize patient benefit.

## 5 Discussion

The current regulatory gap surrounding AI-generated synthetic patient data represents both a challenge and an opportunity for the pharmaceutical ecosystem. Rather than waiting for prescriptive regulations that may lag behind technological capabilities, this manuscript proposes a proactive, principle-based framework to establish trust and enable evidence-based decision-making. By implementing the five principles of Representativeness, Utility, Robustness, Privacy Preservation, and Transparency—operationalized through the Technical Validation Playbook—pharmaceutical companies can build compelling evidentiary dossiers for the acceptance of synthetic patients in regulatory submissions.

### 5.1 Clinical and ethical imperatives for synthetic patient acceptance

Importantly, this framework is not merely an academic exercise but a call to action. We envision this manuscript serving as the catalyst for alignment between industry sponsors and regulatory agencies, particularly the FDA and EMA, in establishing concrete pathways for synthetic patient data acceptance. The stakes are especially high for patient populations facing critical unmet needs, where conventional clinical trial paradigms fail.

Consider the challenge of rare diseases, where patient populations are inherently limited and geographically dispersed. Traditional placebo-controlled clinical trials in these conditions face insurmountable recruitment barriers, forcing companies to abandon promising therapeutic candidates or delay trials for years to achieve adequate enrollment. Similarly, in certain oncology indications with established standards of care, ethical considerations increasingly prohibit true placebo arms, yet single-arm trials lack the statistical power to demonstrate efficacy convincingly.

Synthetic patient data offer a scientifically rigorous and ethically sound alternative. By generating diverse, high-fidelity synthetic populations that capture disease heterogeneity, sponsors can augment insufficient control arms by supplementing limited real-world comparator data with statistically equivalent synthetic patients, thereby enabling adequately powered trials without withholding treatment from additional real participants. Synthetic cohorts can also address ethical constraints by reducing or replacing placebo exposure in conditions where effective therapies already exist, while maintaining statistical rigor through comparators validated against historical real-world evidence. In addition, synthetic populations can democratize access to clinical research by representing demographic groups historically underrepresented in clinical trials—such as racial minorities, elderly patients, and individuals with complex comorbidities—thereby improving generalizability and helping address persistent health disparities. Finally, synthetic patient generation may accelerate precision medicine by enabling the creation of personalized digital twins for rare genetic subtypes where recruiting sufficient real patients is infeasible, supporting targeted therapy development for truly orphan indications.

The regulatory acceptance of synthetic patients in these contexts is not optional—it is imperative. Without validated alternatives to conventional placebo-controlled trials, patients with rare diseases will continue to face treatment deserts, and pharmaceutical innovation in these areas will remain economically unviable.

### 5.2 Limitations and boundary conditions of the proposed framework

This framework carries several limitations that should be acknowledged. First, the proposed principles and the Technical Validation Playbook have not yet been tested through a formal regulatory submission; their practical adequacy therefore remains to be demonstrated through real-world application. Until sponsors submit synthetic patient dossiers structured according to these principles and receive substantive regulatory feedback, the framework retains a normative character that empirical experience may require to be revised. Second, although the quantitative validation methodologies recommended in this work—including specific distance metrics, privacy thresholds, and the TSTR paradigm—draw on established statistical and machine learning practice, the acceptance thresholds appropriate for regulatory decision-making have not been formally calibrated. Metrics such as the 9% disclosure-risk threshold referenced from Health Canada and EMA guidance were developed for the publication of clinical data, not for the acceptance of AI-generated synthetic populations, and their transposition to this context warrants dedicated empirical investigation. Third, this work reflects a single-author perspective informed by industry experience in AI-driven healthcare and synthetic data development; while the manuscript has benefited from expert review, the framework would gain robustness through formal multi-stakeholder validation involving regulatory scientists, biostatisticians, patient representatives, and health technology assessment bodies. It should be noted, however, that the framework's deliberate concentration on structured tabular clinical data reflects the current regulatory reality rather than a methodological limitation. As established in Section 3.3, the electronic case report form remains the definitive evidentiary instrument in regulatory science, and the principle-based framework proposed here is accordingly designed to remain applicable for as long as structured tabular data govern regulatory submissions. Future extensions to additional modalities should be pursued as and when the clinical trial ecosystem evolves to accommodate them.

### 5.3 Toward a new regulatory paradigm

By embracing principle-based validation today, the pharmaceutical ecosystem can unlock the transformative potential of synthetic patients while safeguarding the interests of the real patients whose data underpin these innovations and who ultimately stand to benefit from accelerated therapeutic development.

A natural next step is the development of a formal multi-stakeholder consensus statement; the principle-based framework and Technical Validation Playbook proposed here are intended to serve as a concrete foundation around which such a consensus could be built.

The regulatory science community has historically demonstrated remarkable adaptability in the face of paradigm shifts—from recombinant DNA technology to personalized medicine to adaptive trial designs. Synthetic patient data represent the next frontier. With rigorous validation frameworks and stakeholder collaboration, we can ensure that this powerful technology serves its highest purpose: bringing safe and effective therapies to patients who need them most, particularly those whom traditional trial methodologies have left behind.
